# Interaction ecology and functional stability: a mechanistic framework for managing plant microbiomes in drylands

**DOI:** 10.3389/fmicb.2026.1816170

**Published:** 2026-05-12

**Authors:** Abdul Sami, Wenjuan Mao, Qi Wang, Guo Rui, Qin Xu, Junfeng Wang, Kening Wu, Junfu Li, Lifeng Jiang

**Affiliations:** 1 GreenTech Bank (Shanghai) Agricultural Technology Co., Ltd., Shanghai, China; 2 State Key Laboratory of Efficient Utilization of Agricultural Water Resources, Institute of Environment and Sustainable Development in Agriculture, Chinese Academy of Agricultural Sciences, Beijing, China; 3 School of Land Science and Technology, China University of Geosciences, Beijing, China

**Keywords:** biocrusts, dryland ecosystems, environmental filtering, microbiome assembly, rhizosheath

## Abstract

Drylands are critical ecosystems that support grazing and agriculture, but they are increasingly constrained by environmental stresses such as altered precipitation, warming, salinity/alkalinity, soil pH variability and desertification, which limit plant performance and ecosystem stability. In these conditions, sustaining development through severe, pulse-driven stress is more important for plant “success” than optimizing growth. In addition, soil pH acts as a chemical regulator that modulates microbial activity and resource availability under arid conditions. Through an ecological perspective, this study summarizes how plant-associated microbiomes, particularly bacteria and fungus found in the root and rhizosphere, improve plant performance under arid conditions. Under this filters, microbial communities with functional redundancy, robust interaction networks, and microhabitat-forming characteristics are preferred over single-strain inoculants, which frequently fail under desiccation, UV exposure, temperature extremes, and competition. Key microbial traits, including biofilm formation and extracellular polymeric substances (EPS), contribute to plant resilience under dry conditions. The study further highlights landscape microbial infrastructures—biocrusts and fertility islands—as upstream drivers of microbial source pools and patch-scale resource maps. Finally, it outlines translational priorities for development of stress-adapted consortia and management plans aligned with dryland assembly rules and climate-driven variability.

## 1 Introduction

### 1.1 Drylands as a frontier for plant performance enabled by microbiomes

Drylands are systems where environmental constraints regulate plant–microbe interactions, making them ideal contexts for testing ecological principles of microbiome function. Therefore, drylands are both (i) high-risk areas for plant productivity and restoration failure and (ii) high-opportunity areas where microbiome-based techniques may produce significant marginal gains in soils with limited water and nutrients (Alsharif et al., 2020; Akinmolayan et al., 2025). Plant-associated microbial communities (archaea, fungi, bacteria, and microeukaryotes) can improve plant performance by enhancing water relations, nutrient uptake, disease resistance, and stress signaling.

However, in dry environments, “benefit” must be interpreted as context-dependent: the most beneficial activities are those that stabilize plant growth and development despite heat extremes, saline or alkaline soil chemistry, drought pulses, and persistently low organic matter. This prioritize on stability over maximal growth differentiate dryland systems from mesic ecosystems and places specific demands on both plants and their linked microbiomes. Therefore, arid lands offer not only a test-bed for plant stress tolerance, but also a natural laboratory for identifying stress-tolerant microbiomes and converting ecological principles into microbial solutions for dryland agriculture and restoration (Aslam et al., 2022; Baldauf et al., 2023).

### 1.2 Why “microbial communities” (not just single strains) matter in drylands

Building on this perspective, a crucial question immediately raised when drylands are recognized as opportunity-rich but constraint-dominated systems: what types of microbial interventions can work reliably under such harsh and unpredictable conditions? Early bioinoculant research frequently targeted single plant-growth-promoting bacteria (PGPB) based on characteristics like as phytohormone synthesis, siderophore secretion, phosphate solubilization, or ACC deaminase activity. While such strains can work well under controlled laboratory or greenhouse circumstances, dryland field environments impose steep obstacle, including temperature variations, UV exposure, quick desiccation, irregular wetting, and fierce competition from native microbial communities (Berendsen et al., 2012).

**Figure 1:**
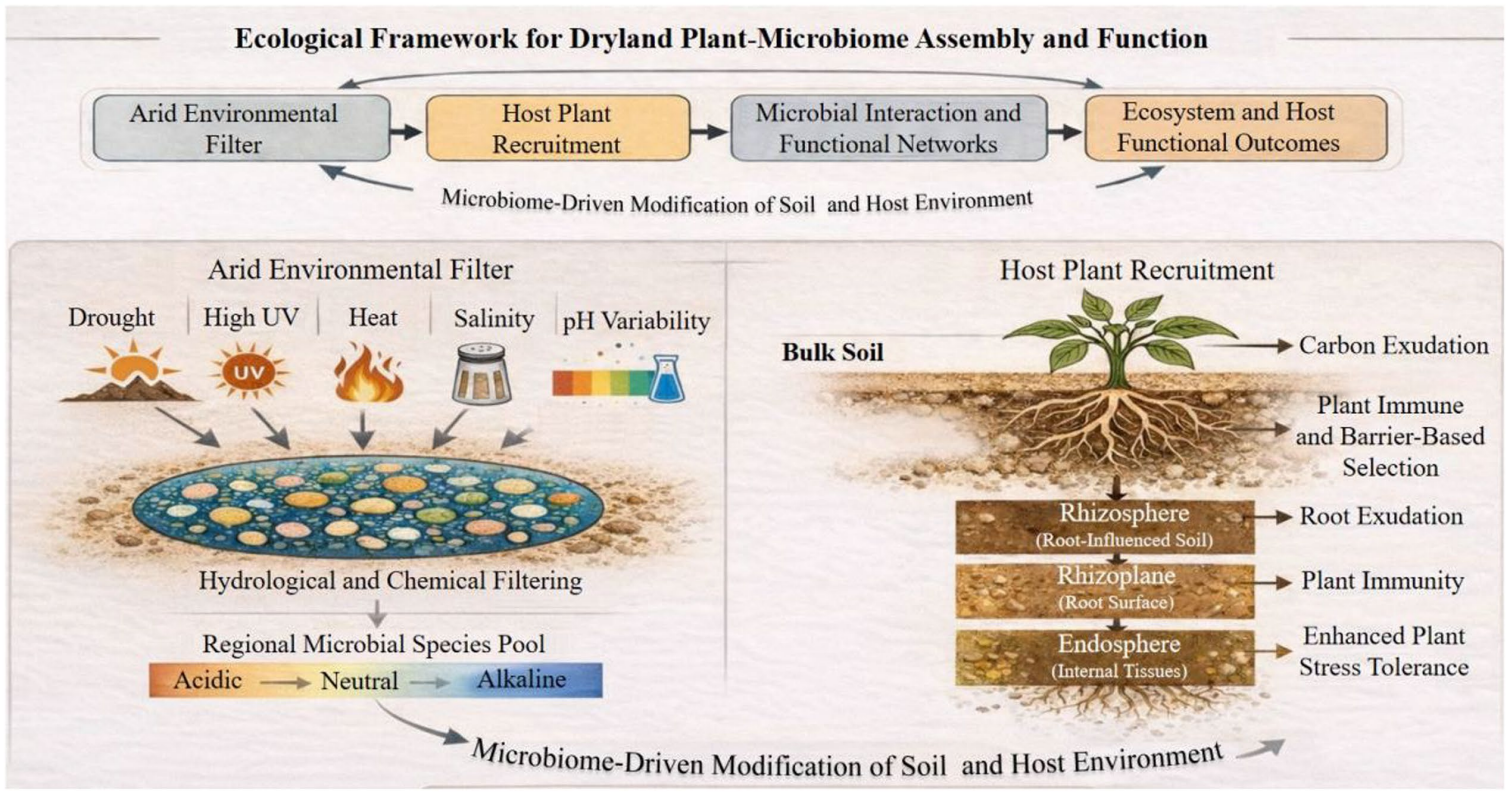
Ecological framework for dryland plant–microbiome assembly and function. Arid environmental filters (drought, heat, UV radiation, pH variability and salinity) shape the regional microbial species pool, followed by host plant–mediated recruitment across bulk soil, rhizosphere, rhizoplane, and endosphere compartments. Microbial interactions and functional networks emerging within these niches drive ecosystem- and host-level functional outcomes, including enhanced stress tolerance. Microbiome-driven modification of soil and host environments generates feedbacks that influence subsequent community assembly and plant performance.

These stresses regularly favor community-level characteristics—functional redundancy, biofilm formation, facilitative interactions, and network resilience—over single-trait excellence (Baldauf et al., 2023; Belnap, 2006; Belnap and Lange, 2013). To put it another way, the very environmental filters that characterize drylands also choose against simplistic, strain-centric solutions. Consequently, the focus of plant microbiome research has shifted from asking “who is present?” to understanding how microbial communities form, endure, and function across plant compartments (rhizosphere, endosphere, rhizoplane, and phyllosphere) and across spatial scales (from root microsites to field mosaics and landscapes) (Figure 1). Extensive synthesis efforts emphasize that predictive microbiome management will necessitate integrating ecological processes—selection, drift, dispersion, and diversification—with reductionist experimentation using synthetic communities, cultures, and mechanistic validation (Belnap and Lange, 2013).

### 1.3 Arid environments amplify the role of interaction ecology

Given the importance of the community-based strategies in drylands, the next question is which characteristics of microbial communities are most important for plant performance under aridity if drylands require microbial communities rather than individual strains. Under strong environmental filtering, interaction networks become primary determinants of microbiome function. Among these traits, biofilm formation and extracellular polymeric substances (EPS) production are particularly important because they enable microbial communities to modify their surrounding microhabitats, thereby influencing water retention, nutrient availability, and root–soil interactions. Importantly, these functions often emerge from coordinated community activity rather than isolated microbial traits. Therefore, interaction ecology becomes fundamental to mechanism: microbial interactions are not only descriptive patterns of co-occurrence, but drivers of functional consequences that directly affect plant growth and development (Berg et al., 2020; Bulgarelli et al., 2012; Bulgarelli et al., 2013).

Therefore, arid ecosystems offer a particularly clear example of a broader principle: under intense environmental filtering, interaction networks control whether microbial populations buffer stress or collapse under it (Figure 1). This realization serves as the foundation for the study’s main hypothesis, which holds that interactions between microbial communities are independent mechanisms that can be purposefully used to improve plant growth and stability in water-limited conditions (Cheraghi et al., 2023).

### 1.4 Scope, definitions, and organizing framework

With focus on ecology, this study builds the understanding of how microbial communities promote plant growth in arid environments, with primary emphasis on ecology, community assembly, and interaction-driven mechanisms. This study primarily focus on (i) plant-associated fungi and bacteria inhabiting roots and rhizosphere soils, (ii) microbial life in key dryland microhabitats—consisting biological soil crusts, “fertility islands,” and rock-associated refugia—and (iii) assembly principles that connect desert ecology with translational microbiome management. This study focuses on compartmental view of the plant microbiome, differentiating between the rhizosphere (soil influenced by roots and exudates), rhizoplane (the root surface), and endosphere (microbes residing within root tissues). This study also take into account dryland-specific microhabitats like biocrusts and rhizosheaths—the soil sheath firmly bound to roots by mucilage and microbial biofilms, which is frequently particularly noticeable under drought circumstances (Bulgarelli et al., 2013; Coleine et al., 2024; Compant et al., 2010).

This study employ a unifying “mechanism spine” based on ecological theory: in arid environments, plant performance arises from a coupled feedback loop wherein environmental filtering shapes host recruitment, host recruitment structures microbial interaction networks, interaction networks determine functional outputs (nutrient, water, and stress regulation), and these results feed back to community assembly.

## 2 Aridity-driven environmental filtering and its effects on the composition of microbial communities in drylands

In dryland ecosystems, aridity serves as a dominate environmental filter that shapes plant-associated microbial communities, resulting in fundamental differences from mesic systems. According to Jansson and Hofmockel (2020) and Coleine et al. (2024), arid and semi-arid environments are characterized by sever water scarcity, dramatic temperature fluctuations, strong solar radiation, frequent salinity and alkalinity stress (Figure 1). Collectively, these elements put intensive selective pressures on soil microbial communities. These conditions favor taxa and traits linked to stress resistance, dormancy, and quick exploitation of transient resource availability. In contrast mesic systems, where moisture and substrate availability permit relatively continuous microbial activity, dryland ecosystems work under a pulse–reserve dynamic, where biological processes are concentrated into short periods after rainfall or irrigation. The microbial taxa that dominate these systems are therefore not necessarily those that grow fastest under ideal conditions, but those that can survive prolonged desiccation, quickly initiate metabolism during brief wet periods, and persist through repeated stress cycles. This fundamental difference in selective regime sets the stage for different community composition and function in arid soils (Bulgarelli et al., 2013; Müller et al., 2016).

Environmental filtering under aridity continually favors microbial traits associated with stress resistance and microhabitat change, such as EPS generation, osmoprotectants synthesis, biofilm formation, robust cell envelopes, and high metabolic flexibility. These characteristics improve survival by maintaining micro-scale hydration, buffering osmotic stress, and protecting against UV light and temperature extremes (Compant et al., 2010; Delgado-Baquerizo et al., 2018). Significantly, such features can directly control plant performance, as biofilms and EPS increase soil aggregation and water retention near roots, while stress-tolerant microbes are more likely to remain active during drought–rewetting periods when plant nutrient demand is high. In dryland soils, aridity also increase spatial heterogeneity, resulting in mosaics of fertile and barren areas like biological soil crusts, rock-associated refugia, and shrub-associated fertility islands, and rhizosheaths. These microsites focus nutrients, moisture, and microbial activity and work as reservoirs that mold the microbial taxa accessible for plant recruitment (De Vries et al., 2020; Duan et al., 2021). Hence, plant–microbe interactions in drylands are extremely context-dependent, due to the spatial structure imposed by aridity and its influence on the assembly and function of microbial community.

## 3 Hierarchical filtering, determinism, and host control in microbe assembly during aridity

Microbial community composition changes predictably with proximity to plant tissues, suggesting a hierarchical filtering cascade from bulk soil to rhizosphere, rhizoplane, and endosphere, according to a robust pattern across plant microbiome study (Figure 1). The main source pool is bulk soil, the rhizosphere is formed by root exudates and changed physicochemical properties, the rhizoplane imposes significant selection related to adhesion and immunity, and the endosphere shows the most rigorously filtered compartment. High-throughput community profiling across model and crop systems and provides strong support for this multi-step selection approach, which serve as a fundamental reference for understanding microbiome assembly in drylands (Coleine et al., 2024; Compant et al., 2010; Edwards et al., 2015).

Environmental constraints exacerbate this assembly cascade in dryland ecosystems. In carbon-poor soil matrices, plant roots serve as uncommon, carbon-rich oases that provided sharp temporal and geographical variations in microbial growth potential. Rhizosphere communities can be notably enhanced by root exudation, but recruitment is limited by short moisture windows, disrupted connection, and desiccation stress. As a result, timing in relation to irrigation pulses or rainfall becomes an important but studied aspect of microbiome assembly in arid environments (Alsharif et al., 2020; Costa et al., 2018). Thus, the effects of both deterministic and contingent processes are amplified in plant-associated microbiomes in drylands because they are “doubly filtered,” first by host-mediated selection and subsequently by harsh environmental circumstances.

Microbiome assembly is often thought of as the result of dispersal limitation, stochastic drift, environmental and host-driven deterministic selection, and historical contingency. Drylands exemplify systems in which strong selection (heat, salinity, drought, UV radiation) coexist with strong dispersal constraints imposed by habitat patchiness and poor moisture connectivity (Figure 1). At fine spatial scales, root exudates and plant immune consequence exert strong deterministic control over recruitment, whereas at larger spatial and temporal scales, dust transport, dispersal limitation, episodic disturbances, and rainfall pulses can reset communities and introduce stochasticity (Cheraghi et al., 2023; Costa et al., 2018; Fang et al., 2025). This paradigm explains why similar plant species can host divergent microbiomes across sites and why microbial inoculants may succeed in some dryland contexts but fail in others.

The reorganization of root-associated bacterial communities under water limitation, which frequently involves the enrichment of drought-tolerant lineages like Actinobacteria, is a drought-associated phenomenon that is frequently seen across plant hosts. This phenomenon has been synthesized across systems and associated with host-mediated modification in exudation, root features, environmental filtering and immunological signaling (Fitzpatrick et al., 2020; Hardoim et al., 2008). These processes are interdependent: drought-induced changes in plant physiology feed back on microbial metabolism, and microbial activity can affect how plants react to stress (Fitzpatrick et al., 2020; Hardoim et al., 2008; Hartmann et al., 2021). Plant drought strategies are therefore active elements of the microbiome building process rather than just background factors.

Host features play a pivotal role in regulating persistence and recruitment. In drylands, plants apply disproportionate control over microbial communities because they provide some of the only predictable carbon inputs into severely carbon-poor soils. Root architecture traits that improve water capture—such as deep rooting, fast fine-root proliferation after rainfall, and rhizosheath production—also control where and when carbon is released, shaping microbial hotspots. Tariq et al. (2024) indicates how desert root systems modulate carbon and nutrient accumulation across soil profiles, implying corresponding vertical and lateral structuring of microbial community composition and function. Plant immune recognition and compartment boundaries further limit which microbes can persist near or inside roots, making compartment-resolved sampling crucial for identifying reliable, functionally relevant symbionts (Compant et al., 2010; Hassani et al., 2018).

Importantly, host filtering is dynamic rather than static. Plant phenology and pulse timing can decide whether beneficial microbes establish early enough to influence subsequent drought responses since recruitment chances in drylands are frequently concentrated into brief wet periods. This pulse-driven assembly helps illustrate why drought-adapted plant microbiomes may show comparatively limited taxonomic diversity but big functional stability, as community composition is constantly reassembled under predictable stress regimes (Havrilla et al., 2019).

### 3.1 Community interactions, stability, and dryland microbiome engineering implications

Although environmental and host filtering determine which microbes assemble, an equally important question is how these microbes interact once established. In dryland root areas, resources come as pulses, generating boom–bust dynamics that promote niche partitioning, cooperation, and facilitation. Microbial interactions can alter realized niches through cross-feeding, competition for limited nutrients, antagonism, and cooperative activities like EPS production and biofilm formation that regulate microscale moisture conditions. An apparent paradox is resolved by these interaction-mediated processes: despite strong environmental filtering and reduced diversity, dryland plant microbiomes can retain their functional capacity under long-term stress. Network-based perspectives highlight the importance of community structure—rather than the existence of specific taxa alone—plays a key role in determining microbiome stability and functional consequences. Communities can be protected against environmental changes and taxonomic turnover by “hub” taxa, redundancy among functional groups, and facilitative interactions (Delgado-Baquerizo et al., 2018; Fang et al., 2025). In arid systems, Lan et al. (2024) further underscores the diversity of dryland microbial adaptations and the vulnerability of interaction networks to desertification and climate change, processes that can modify ecosystem functions and communities.

Acknowledging the significance of interaction ecology shifts the emphasis from “who assembles” to “how communities persist and function.” This transition provides a link applied translation and mechanistic understanding. From an engineering perspective, assembly ecology suggests that effective microbiome interventions need to either intentionally alter filters by altering host characteristics or soil conditions, or introduce microbes that can pass both environmental and host filters and integrate into existing networks. This strategy, which combines ecological theory with translational tools like cultured isolates, synthetic communities, and field validation, is demonstrated by Li et al. (2024) as rational microbiome management.

Identifying stress-adapted source pools, such as desert soils, biological soil crusts, and rhizosheaths; quantifying assembly rules across compartments and time, especially in relation to moisture pulses; and generating microbial consortia with interaction structure in mind, emphasizing facilitation for establishment, redundancy for stability, and minimal antagonism, become several priorities for dryland applications (Cheraghi et al., 2023; Hestrin et al., 2019).

## 4 Water economy and hydraulic buffering: EPS–biofilms, mucilage biogels, and rhizosheath microrefugia

**Figure 2:**
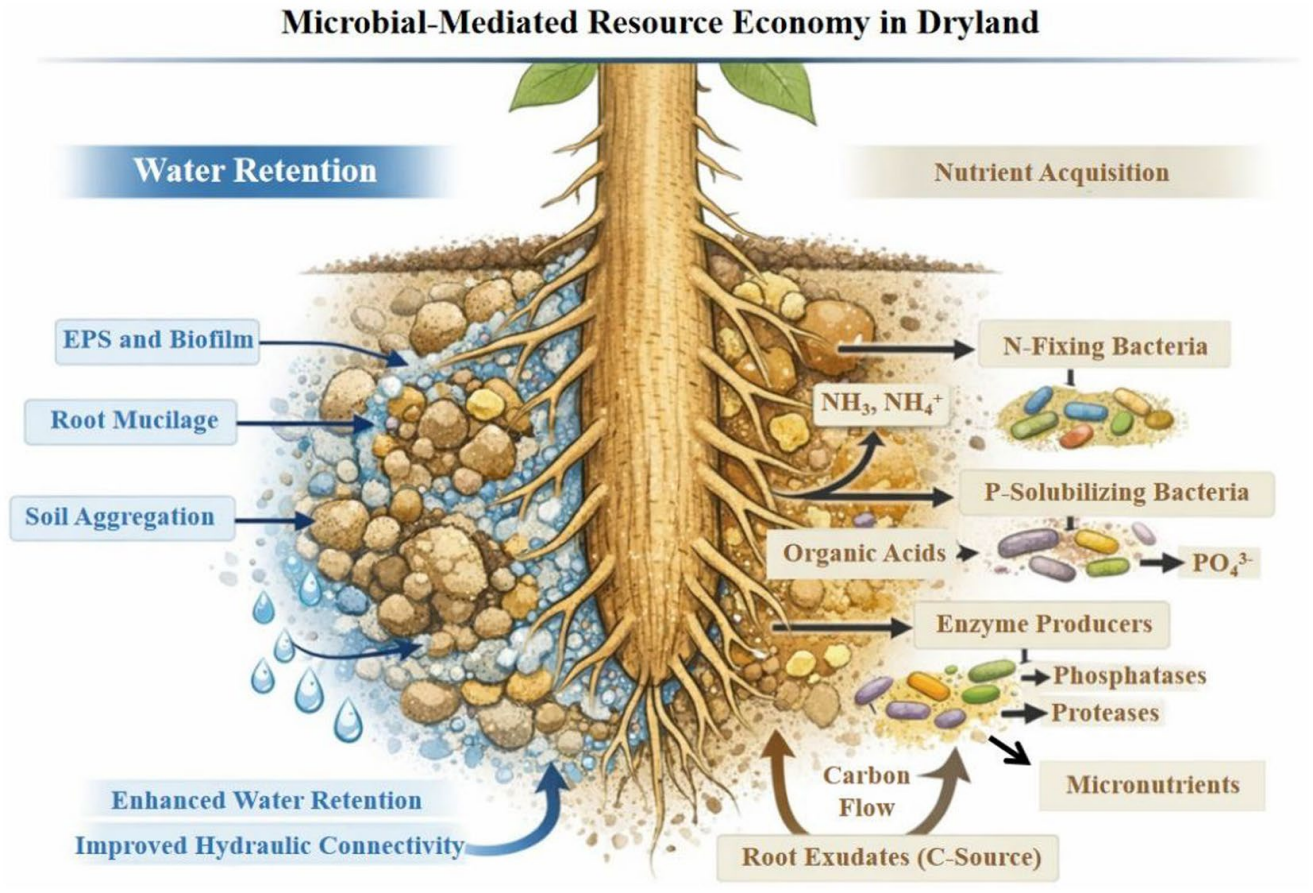
Microbial-mediated resource economy in dryland soils. Plant–microbe interactions regulate water retention and nutrient acquisition under dryland conditions. Microbial extracellular polymeric substances (EPS), biofilms, root mucilage, and soil aggregation enhance soil water retention and hydraulic connectivity. Root-derived carbon exudation fuels microbial processes such as nitrogen fixation, phosphorus solubilization, enzymatic mineralization, and micronutrient mobilization, collectively supporting plant nutrition and growth in resource-limited soils.

Beyond community assembly and interaction dynamics, dryland conditions impose rhizosphere-scale physical constraints in which thin water films collapse, diffusion slows, and roots experience rapid hydration–desiccation cycles. Meanwhile, microbes face osmotic stress, sever competition and hunger during short wet pulses. Microbes that hold water locally, stabilize soil structure, and preserve root-soil connectivity can produce hydraulic buffering that prevail beyond the wet pulse, making microhabitat change a key pathway to drought advantage in these circumstances (Figure 2). According to dryland syntheses, features that provide microbial resistance to climate stress and desertification also stabilize microsites. When these qualities are recruited to roots, they serve as extensions of plant drought strategies (Hu et al., 2024).

Microbial EPS generation and biofilm synthesis provide a central mechanism of hydraulic buffering in drylands. EPS are high-molecular-weight polymers that bind microbial cells to soil particles and to each other, forming structured matrices that enhance aggregation, water retention, and microscale hydration. A previous study identify various drought-relevant effects, including improved particle cohesion, increased microscale water retention through hygroscopic characteristics, and generation of chemical microenvironments that buffer fast changes in water potential (Hu et al., 2024; Jansson and Hofmockel, 2020). Mucilage and microbial EPS are progressively described as functionally equivalent biogels that modify viscosity, water-film connectivity, and diffusion pathways in rhizosphere biophysical formation. A synthesis treating mucilage as a biofilm-like matrix argues that shared chemical and physical characteristics with microbial EPS support maintain interfacial continuity and prolong solute transport as soils dry. This results in a plant-microbe synergy whereby microbes contribute EPS, biofilm architecture, and community stabilization, while mucilage provides a carbon-rich scaffold. Together, these factors prolong root–soil contact and postpone hydraulic stress at the root surface (Jansson and Hofmockel, 2020).

The rhizosheath, the soil layer firmly attached to roots by mucilage, microbial biofilms, and root hairs, is where these EPS–mucilage interactions are most noticeable. Studies focusing on rhizosheaths characterize it as a chemically unique nich and physically coherent environment that is rich in microbes that are directly related to water relations. By lowering evaporative loss, stabilizing the root–soil interface against shrink–swell cycles, and preserving a habitat where microbial activity continues during drying, the rhizosheath functions as a desiccation buffer. Improved water-use efficiency under drought and irrigation-driven changes in rhizosheath microbiomes are reported in empirical investigations on crops like rice, suggesting that water management can partially modify community composition and function (Lan et al., 2024). This niche in dryland context is consistent with more general evidence that microrefugia shape desert microbiomes and that microbial activity is concentrated in plant-associated habitats in harsh environments (Costa et al., 2018).

Therefore, hydraulic buffering is not just a reflection of polymer production but also of community organization. EPS is often a shared resource, and biofilms are interactive systems where matrix stability is determined by spatial organization, cooperation, and division of work. Soil biofilm syntheses exhibit that biofilm persistence improves aeration, aggregate stability, and water retention, with plant advantages rising at the community level rather than from individual strains. For drylands, this implies that microbiomes enhancing water relations are those that incorporate into resident communities and form stable interfacial matrices during short wet windows (Belnap and Lange, 2013).

### 4.1 Pulse-driven microbial control of nutrient availability in drylands

Nutrient limitation in arid conditions results from a combination of chemical immobilization, limited movement under low soil moisture, and low biological inputs. Alkaline and carbonate-rich environments further decrease phosphorus (P) availability through precipitation and adsorption, whereas drying soils severely restrict the diffusion of nitrate, phosphate, and micronutrients. Low organic matter constrains enzymatic mineralization and microbial turnover. Thus, chemistry, transport, and timing all play a role in nutrient intake, with brief wet pulses producing limited windows for mobilization and absorption. Recent studies indicate how spatial variability and root strategies structure carbon and nutrient accumulation, connecting microsite availability and nutrient dynamics to root architecture (Akinmolayan et al., 2025; Lan et al., 2024). In nutrient-poor drylands, biological nitrogen fixation (BNF) can play a significant role, especially in hotspots like rhizospheres, biocrusts, and fertility islands (Figure 2). Nevertheless, the context of the microhabitat is crucial since fixation requires energy and is sensitive to dryness. Niche-based syntheses underscore the importance of habitat structure and temperature restrictions by demonstrating that fixation rates depend on diazotroph community composition and niche breadth rather than nif gene presence alone (Li et al., 2024; Wang H. et al., 2025).

In symbiotic systems, drought can inhibit nodulation and fixation by reducing carbon availability and changing root physiology; experimental studies exhibit coordinated change in fixation, root characteristics, and microbiomes under water shortage. These findings imply that when diazotrophs continue to exist in buffered root-associated microsites and plant carbon allocation stays constant over drying cycles, nitrogen advantages are most likely to occur. This suggests that, from the standpoint of dryland management, nitrogen benefits are most likely when microbial communities are sustained by stable root-associated microhabitats (such as rhizosheaths) and by plant carbon allocation patterns that endure throughout drying cycles, as opposed to depending on fixation, which breaks down as soon as soils dry (Lan et al., 2024).

In arid and semi-arid soils, especially in alkaline or calcareous conditions, phosphorus (P) and potassium (K) availability is frequently constrained. Microbial P mobilization take place through multiple, complementary processes: (i) acidification and organic acid production that dissolves mineral P and chelates cations; (ii) emission of phosphatases and other enzymes that mineralize organic P; and (iii) production of compounds (including EPS and siderophores in certain conditions) that affect P binding and availability. Mechanistic syntheses identify organic-acid-driven pathways as dominant for inorganic P solubilization and elucidate underlying enzymatic and genetic processes (Li et al., 2024; Li et al., 2025). Potassium (K) availability is also constrained in drylands, not by total abundance but by limited diffusion and strong fixation to clay minerals under low soil moisture. Microbial processes can enhance K accessibility through organic acid production, mineral weathering, and EPS-mediated maintenance of hydraulic connectivity, with short wet pulses enabling transient release and root interception.

Drylands add a significant limitation: P solubilization is frequently most successful during short hydration periods when solute transport toward roots is feasible and microbial metabolism, acid secretion, and enzyme function are active (Figure 2). This pulse dependence implies that community-level organization can regulate whether the benefits of P translate into plant uptake: consortia that combine fast responders (quick acidification), enzyme generator (organic P mineralization), and interface stabilizers (EPS/biofilms that sustain micro-scale hydration and keep mobilized P near roots) should, in principle, outperform single-trait strains. This “division of labor” logic aligns with broader calls for rational microbiome management that integrates ecology, assembly constraints, and mechanistic validation instead of addressing each characteristic separately (Liu et al., 2024). Iron (Fe) is frequently found in insoluble forms, Fe limitation can take place even in soils that are rich in minerals. Microbial siderophores—high-affinity Fe-chelating molecules—are an important pathway by which plant-associated microbes can affect iron availability in the rhizosphere, particularly under alkaline environments where solubility of Fe is low. Significantly, siderophore pathways also interact with plant defense: beneficial bacteria can lower the availability of Fe to pathogens and aid in competitive exclusion in the rhizosphere by sequestering Fe. In dryland, where nutrient scarcity amplifies competition, siderophore-mediated techniques may therefore assist both nutrition and disease suppression, again supporting the thought that nutrient economy processes are integrated in interaction ecology instead of being separate “add-ons.” Micronutrients including zinc (Zn), manganese (Mn), and copper (Cu), whose availability is decreased in alkaline, carbonate-rich soils as a result of precipitation and adsorption, exhibit similar pulse-dependent dynamics. Microbial chelation, localized acidification and redox transformations can temporarily mobilize these elements during hydration events, while biofilms and rhizosheath structures help retain mobilized ions near roots. Together, these processes support the idea that micronutrient acquisition in drylands is controlled by microbial community organization, microsite buffering, and synchronization with episodic water availability instead of bulk soil nutrient pools alone.

### 4.2 Signaling, cellular stability, and interaction ecology under drought

In addition to hydraulic limitations, drought presents a signaling problem that requires plants to balance resource allocation, defense, and development under varying carbon supplies. Many microbiome benefits under aridity arise because microbes influence this choice space, either by changing hormone pools or by rearranging signaling networks so that drought outcomes are expressed more expeditiously. This logic consistent with study that drought resilience appears from coupled plant–microbe feedbacks that become functionally significant during and after stress periods (Liu et al., 2024; Naylor et al., 2017). One well-characterized example is 1-aminocyclopropane-1-carboxylate (ACC) deaminase. ACC-deaminase-producing bacteria lower stress-induced ethylene accumulationby breaking down ACC (the ethylene precursor), which limit root growth during drought. Grapevine rhizosphere isolates, such as *Pseudomonas*, *Enterobacter*, and *Achromobacter*, provide a clear example of an arid zone. In pot experiments, these strains enhanced grapevine performance under drought by combining ACC deaminase activity with other PGP traits (IAA, EPS, P solubilization, and N fixation) (Lundberg et al., 2012). Increasingly, evidence indicates that ACC deaminase is most efficient when combined with characteristics that promote microbial determination and nutrient delivery at the root interface (Aslam et al., 2022).

**Figure 3:**
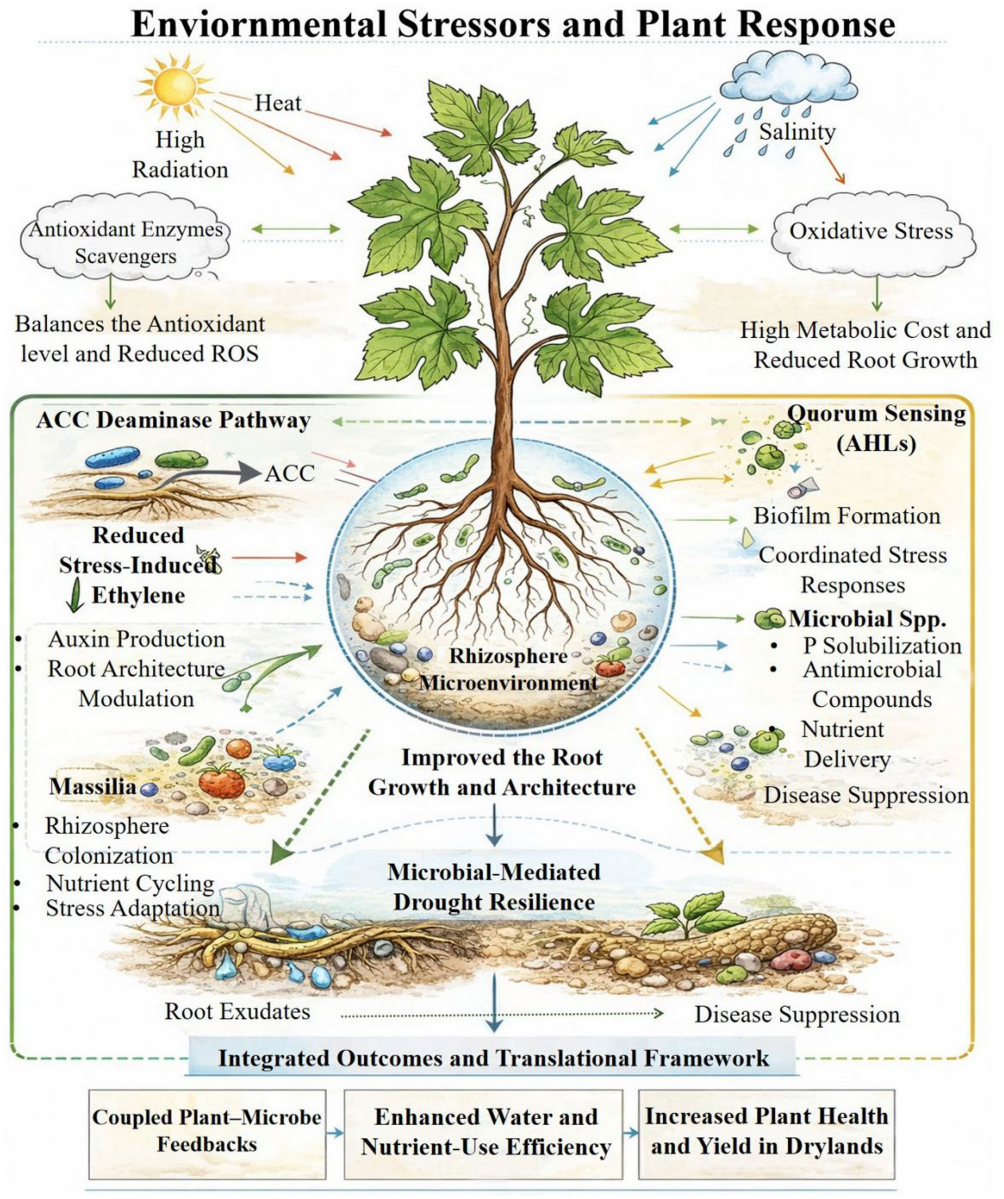
Microbial pathways linking environmental stressors to plant stress tolerance. Abiotic stressors, including high radiation, heat, and salinity, induce oxidative stress and constrain root growth in dryland plants. Plant-associated microbes mitigate these effects through antioxidant regulation, ACC deaminase–mediated reduction of stress-induced ethylene, quorum sensing, and functional traits related to nutrient acquisition and biocontrol. These coordinated microbial processes improve root architecture and enhance plant drought resilience.

A parallel mechanism is represented by the generation of microbial auxin (indole-3-acetic acid, IAA), which expands the spatial footprint of water seeking and modifies root architecture (Figure 3). In arid conditions, this relates microbial metabolism to root placement and timing instead of just biochemical tolerance. A common synergy is suggested by the fact that many drought-effective isolates co-express IAA production with EPS/biofilm traits. IAA support in the development of more absorptive root surface area and root hairs, while microbial EPS stabilizes the hydrated microenvironment surrounding those structures, increasing the probability that a brief pulse will result in significant water uptake (Lundberg et al., 2012).

Additionally, hormone-associated chemistry and root exudation are altered by drought. In spring wheat, multi-omics analyses revealed drought-induced enhances in sugars and ABA in exudates that correlated with enrichment of drought-beneficial taxa, framing recruitment as a correlate “self-rescue and cry-for-help” plan (Marasco et al., 2012). Quorum-sensing N-acyl-homoserine lactone (AHLs) can prime plant responses, boosting tolerance to drought and salinity without persistent defense costs, which is another way that microbial signaling molecules support in stress preparedness (Marasco et al., 2018).

A defining characteristic of arid stress is that drought often acts alone; high radiation, heat, and salinity frequently coexist and exacerbate oxidative stress, membrane damage, and protein instability. In response, plants produce non-enzymatic scavengers and antioxidant enzymes, although these defenses are metabolically costly and may not be sufficient during repeated stress cycles. Microbial partners can increase tolerance through two complementary ways: (i) Osmotic adjustment, in which compatible solutes stabilize proteins and membranes under low water potential, and (ii) ROS management, in which microbial association elevates the plant’s antioxidant capability or decrease ROS production by stabilizing physiology (Liu et al., 2024). A keystone *Massilia* strain altered root osmotic and immunological gene expression, including ROS scavenging pathways, across *Arabidopsis* ecotypes, suggesting that oxidative buffering may also take place via microbiome-mediated reprogramming (Mourouzidou et al., 2025). Together, these mechanisms highlight that drought tolerance emerges from coordinated community processes rather than isolated traits (Costa et al., 2018).

### 4.3 Defense, disease risk, and interaction ecology as a framework for translation

Drought modifies the dynamics of disease in a variety of ways, sometimes decreasing infections but frequently raising susceptibility due to compromised host defenses and altered resident microbiota. Therefore, microbiomes that promote growth in arid environments must also reduce biotic risk. Through competitive exclusion, antimicrobial production, and immunological priming, broad syntheses connect microbiome assembly to plant health. These effects become particularly significant when abiotic stress reduces plant recovery capacity (Fang et al., 2025). Priming-based defense, sometimes referred as induced systemic resistance, enables plants to react more quickly to biotic and abiotic stresses. Quorum-sensing AHLs emphasize chemical communication as a pathway for whole-plant resilience by offering a clear mechanism via which microbial signals generate systemic tolerance to drought and salinity (Marasco et al., 2018). In drylands, where stress is common and uncertain, such priming processes are especially important because they increase response efficiency rather than requiring continuous defensive investment.

**Figure 4:**
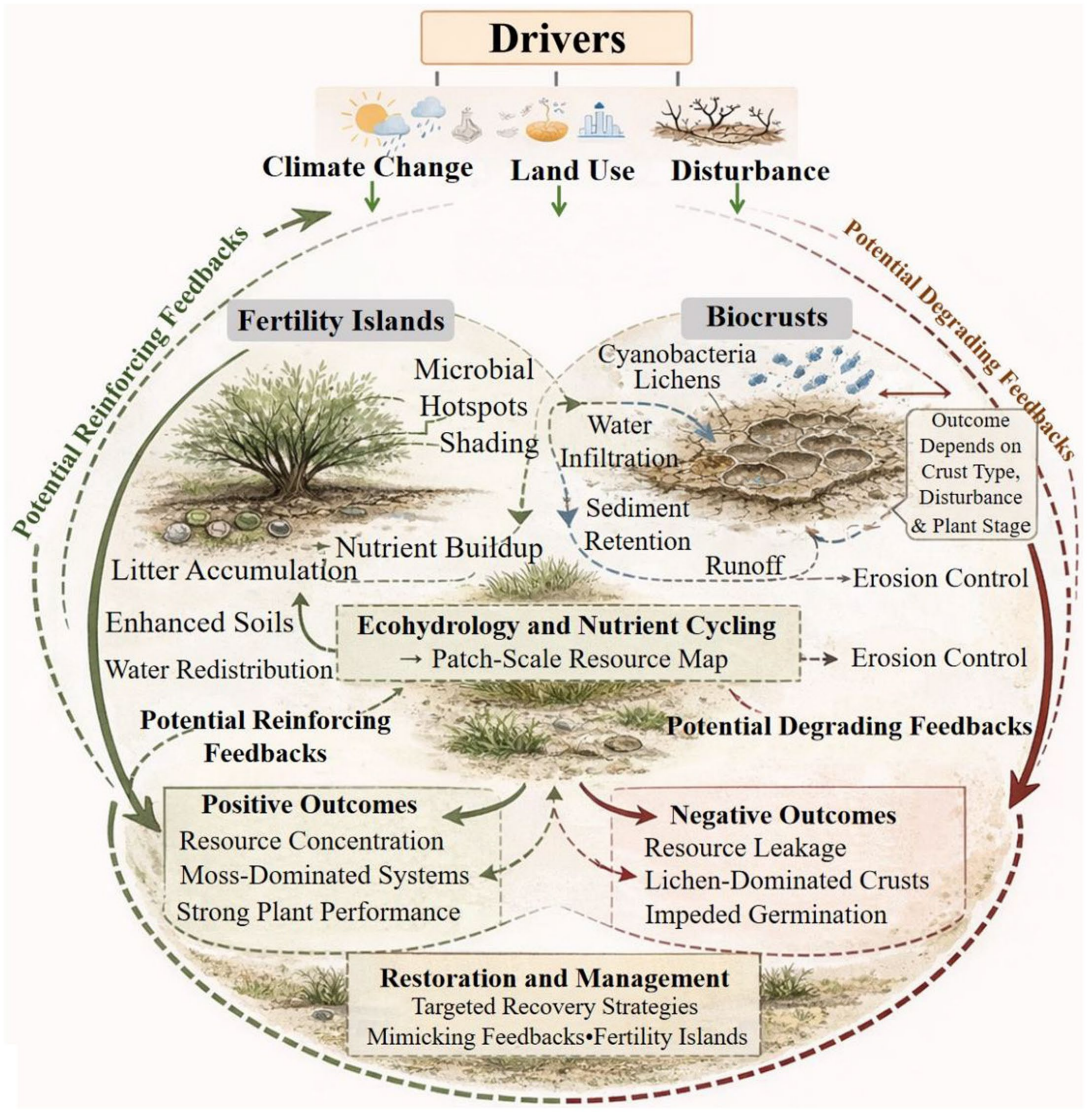
Feedback-driven ecohydrological processes shaping dryland ecosystem outcomes. Climate change, land use, and disturbance interact with fertility islands and biological soil crusts (biocrusts) to regulate patch-scale ecohydrology and nutrient cycling in drylands. Reinforcing feedbacks promote resource concentration, plant performance, and ecosystem stability, whereas degrading feedbacks lead to resource leakage, erosion, and impaired germination. Restoration and management strategies that mimic natural feedbacks, such as fertility island formation, can redirect dryland ecosystems toward sustainable trajectories.

In arid soils, disease control is highly dependent on interactions (Figure 4). Insufficient moisture increases competition for carbon, attachment sites, and micronutrients, enabling beneficial bacteria to control diseases through resource sequestration and niche preemption. Consequently, stress-tolerance characteristics (EPS, osmolytes) and antagonistic abilities (siderophores, antimicrobial synthesis) are frequently combined in drought-effective consortia (Mourouzidou et al., 2025). The key implication is that growth promotion and disease control should be integrated goals in dryland systems, where drought reduces the margin for error. Numerous processes related to drought exhibit shared or community-level characteristics, rely on host recruitment signals, and need to be compatible with resident microbial networks. Mechanistic data from *Arabidopsis* and wheat demonstrates that functional outcomes are more reliably governed by drought-driven changes in exudation and community composition, as well as keystone taxa and network structure than single traits (Marasco et al., 2012; Mourouzidou et al., 2025). According to Marasco et al. (2012) and Li et al. (2025), translational frameworks place a strong emphasis on integrating ecological knowledge with causal validation to generate microbiomes that endure, integrate, and function in dryland environments.

## 5 Surface and canopy microbial infrastructures that influence dryland “resource maps”: fertility islands and biocrusts

Plant performance in many drylands is controlled by both root-proximal processes and landscape “infrastructures,” that determine where conducive root environments exist. Moreover, arid ecosystems are characterized by intense resource limitation and strong spatial heterogeneity, nutrients and water are often distributed equally. Rather, drylands function as a patchwork of biologically active areas and relatively arid places, forming “resource maps” that have a significant impact on plant establishment, survival, and productivity. Shrub-driven fertility islands and biological soil crusts (biocrusts) are two predominant structures that provide enduring microhabitats that control microbial source pools, nutrient buildup, and water routing. In parallel, biocrusts—cohesive surface communities made up primarily of cyanobacteria, lichens, mosses, fungus, and heterotrophic bacteria—stabilize sediments and control biogeochemical cycling and ecohydrology in drylands with little vegetation (Nazari et al., 2022; Naylor and Coleman-Derr, 2018).

In the upper surface of soil, biocrusts are cohesive surface communities that are dominated by photoautotrophs (algae, lichens, cyanobacteria, and mosses) entwined with heterotrophic bacteria, fungi, and archaea. Biocrusts serve as a dominating biological ground cover that stabilizes sediments and controls near-surface ecohydrology and biogeochemistry in areas with limited vascular plant cover (Figure 4). Large-scale syntheses highlight that they strongly impact first-contact mechanisms during rainfall pulses including whether water runs off or infiltrates, whether nutrients are lost to erosion or retained, and whether seedlings establish on moving substrates or stable microsites. According to Nazari et al. (2022), these syntheses also emphasize vulnerability of biocrusts disturbance and climate fluctions: warming and altered precipitation regimes, can weaken nutrient cycling at regional scales, increase dust emissions, and decrease biocrust cover and function.

A parallel canopy-associated infrastructure is represented by fertility islands. Shrubs and perennials produce under-canopy patches in many drylands that are distinguished from bare interspaces by higher organic matter, moderated microclimates, enhanced nutrient concentrations, and increased microbial biomass and diversity. Woody plant encroachment, erosion, and nutrient redistribution can produce stable patterned landscapes with resource-rich shrub islands encircled by nutrient-poor interspaces (Newton et al., 2010). In highly alkaline arid desert higher macro- and micronutrients beneath shrubs, increased bacterial and fungal diversity, and enrichment of taxa including rhizobia and Glomeraceae. Likewise, research in China’s deserts demonstrate that fertile-island intensity (frequently linked to canopy size and litter interception) structures soil microbial community composition and function, emphasizing shrub architecture and canopy microclimate as upstream drivers of microbial assembly (Philippot et al., 2013). Fertility islands also generate specific niches; for instance, halophyte shrubs may produce saline fertile islands that select for microbial energy-use approaches adapted to aridity and salinity simultaneously, changing the microbial pool accessible to subsequent recruits (Raaijmakers and Mazzola, 2012).

### 5.1 How patches control plant-available resources

Dryland hydrology is pulse-driven and spatially unequal, biocrust value lies less in “adding water” than in controlling where water moves, how long it is accessible, and how much soil is lost during the pulse. A fundamental synthesis highlights that hydrologic results rely on crust kind (cyanobacterial vs. lichen- vs. moss-dominated), soil texture and disturbance; biocrusts can either improve infiltration or enhance runoff, while also controlling sediment formation and redistribution. Whether fertile microsites endure long enough to promote plant establishment is directly determined by these mechanisms (Rawat et al., 2021; Ridolfi et al., 2008). This reasoning is further extended to larger scales through modeling. According to spatially explicit ecohydrological simulations, biocrusts effectively concentrating water into vegetated patches and change local water availability to plants outside of the crust patch itself by intensifying surface water redistribution and rearranging runoff–runon dynamics (Rillig et al., 2019). Thus, biocrusts function as key regulators of dryland water routing rather than uniform enhancers of soil moisture.

In environments where below ground inputs are limited by low organic matter and sporadic microbial activity, surface microbial populations also influence nutrient availability. Since many arid soils are nitrogen-limited and pulses of available nitrogen may coincide with brief windows for root uptake, evidence continues to support the idea that biocrusts affect nitrogen transformation pathways (including mineralization and nitrification) (biocrust nitrogen transformation research). Together, nutrient cycling and biocrust hydrology produce a patch-scale “resource map” in which water routing, nutrient timing and erosion control jointly determine where the formation of safe sites and how long resource reservoirs persist.

However, because biocrust-plant interactions are highly context sensitive, plant outcomes are not always favorable. A global meta-analysis found no single overall direction of effect, displaying that consequences depend on biocrust composition, plant characteristics, and ontogeny. Moreover, establishment is sensitive to surface roughness, infiltration patterns, and mechanical barriers, seedlings may react differently than adults to moss-dominated crusts, which more frequently promote plant performance whereas lichen-dominated crusts can impede it (Rodriguez-Caballero et al., 2018). Biocrusts can assist plants by stabilizing soil, decreasing erosion, and prolonging resources, but can also suppress germination or emergence by hardening the surface, changing albedo/temperature, or shifting infiltration in ways that decrease seed–soil contact under specific conditions (Rawat et al., 2021; Rodriguez-Caballero et al., 2018). This conditionality is a fundamental principle for understanding how microbial landscapes affect plant performance and determining whether interventions should focus on longer-term nutrient accumulation, erosion management, or seedling establishment.

### 5.2 Feedbacks between plants and microbial patches

A systematic foundation for relating patch-scale soil change to plant community outcomes is provided by plant–soil feedback (PSF) theory. PSFs explain how plants change the biotic and abiotic characteristics of the soil and how such changes affect the performance of the same species or other species. In drylands, PSFs are particularly consequential because minor changes in microbial composition, soil structure, or nutrient availability can push establishment above or below survival thresholds. Conceptual syntheses emphasize that feedbacks may be either positive (reinforcing dominance and patch formation) or negative (promoting coexistence), and that altering microbial communities, pathogen pressure, and mutualist effectiveness, shifting stress regimes and disturbances can change the strength of feedbacks and even reverse their sign (Rosenberg and Zilber-Rosenberg, 2019; Schlaeppi and Bulgarelli, 2015; Singh et al., 2022).

According to Ridolfi et al. (2008) and Liu et al. (2024), fertility islands can be understood as positive PSFs at the patch scale: shrubs alter soil through litter accumulation, moisture retention and shading; these modifications select microbial communities with improved nutrient cycling and possibly higher mutualist availability; enhanced soils then favor shrub persistence and recruitment, reinforcing spatial patterning. When restoration tries to encourage specific native species or when microbial supplements run the danger of promoting unwanted vegetation, negative PSFs can also arise through pathogen buildup or resource depletion, impacting succession, limiting local dominance. This PSF lens also elucidates how climate change functions as a feedback modulator. The same pressures that endanger dryland microbiomes—warming, changed precipitation, and desertification—can loosen stabilizing structures like biocrusts and move soils toward erosion-driven states that export nutrients and decrease microbial function. According to Zhang et al. (2018), syntheses highlight that reductions in biocrust cover and function might increase dust emissions and interfere with nutrient cycling, changing the initial soil state that seeds and roots encounter. According to Zhao et al. (2024), dryland microbial syntheses emphasize that desertification and climate stress have the potential to reorganize microbial communities, which in turn can alter the direction and intensity of plant–soil feedback loops. Taken together, these ideas motivate considering dryland management as feedback management rather than only input management.

### 5.3 Patch-based strategies for restoration and agriculture

A key translational implication is that drylands function as patch systems, thus microbial strategies that promote plant growth must be assessed and implemented at the patch scale rather than based on site averages. In order to improve the permanence of resource reservoirs and promote safe-site availability, biocrust recovery or rehabilitation can stabilize soil, lessen erosion, and rearrange water redistribution. While acknowledging that certain islands may impose salinity-linked constraints depending on shrub identity, shrub-associated fertility islands can be protected or purposefully mimicked to generate microbial and nutrient hotspots that improve seedling establishment and early growth (Raaijmakers and Mazzola, 2012).

According to Van der Putten et al. (2013), PSF theory provides a useful guide for timing and placement: interventions can be staged to strengthen desired positive feedbacks (such as soil stability and nutrient retention supporting recruitment) and avoid pushing systems into erosion-amplifying negative feedbacks under drought. Additionally, this patch-first framework also complements rational microbiome management techniques that prioritize matching interventions to ecological filters and assembly rules, establishes a clear scaling principle for drylands: successful plant–microbe partnerships frequently depend on where establishment occurs and which microhabitat legacies (biocrust-conditioned surfaces or canopy-conditioned island soils) govern resource timing and microbial source pools (Rodriguez-Caballero et al., 2018; Tariq et al., 2024). This means that rather than being delivered as uniform treatments, microbial interventions may need to be spatially targeted (to safe places, crust states, or canopy patches) and temporally staged (to fit successional trajectories and life-stage sensitivity).

## 6 Climate change and land use as co-drivers reshaping dryland plant–microbe systems

Building on these ecological and landscape-level processes, climate change acts as a major co-driver in drylands by intensifying existing constraints such as higher temperatures, prolonged droughts, variable rainfall, and more intense precipitation events. This amplification modifies the timing and strength of the ecological filters that already shape microbial communities in arid environments for plant-microbe systems. Broad soil microbiome syntheses demonstrate that climate change affects microbial activity, diversity, and biogeochemical roles through moisture and temperature effects, with results strongly mediated by vegetation and soil characteristics (Alsharif et al., 2020; Tariq et al., 2024). Even slight modifications to precipitation patterns can reorganize microbial systems supporting nutrient cycling, soil structure, and plant performance in arid regions where water availability already dominates ecosystem behavior (Li et al., 2025). Increased rainfall variability, which is frequently defined by fewer but more severe precipitation episodes, is a defining characteristic of climate change in drylands. This change favors microbial taxa capable of fast stimulation during pulses and prolonged survival during dry intervals, potentially strengthening selection for stress-tolerant and fast-responding guilds. The integrity of important microbial habitats including biocrusts, rhizosheaths, and fertility islands can be compromised by heavy rains, which can also enhance erosion and nutrient leaching (Figure 4). This generates a paradox: stronger pulses may temporarily boost microbial activity and nutrient availability, while simultaneously destabilizing the biological and physical infrastructures that support beneficial plant–microbe interactions across seasons (Akinmolayan et al., 2025).

These dynamics are further altered by warming, which affects microbial metabolism during short wet spells. Although higher temperatures normally accelerate microbial activity, under aridity this response is limited by water scarcity, leading to nonlinear consequences. Experimental and modeling studies show that warming may enhance respiration and carbon turnover during wet phases while suppressing activity during prolonged dry periods, potentially reducing soil carbon stocks and shifting communities toward survival-oriented strategies (Alsharif et al., 2020; Tariq et al., 2024). Warming can also reorganize microbial communities in favor thermally tolerant taxa, frequently overlapping with drought-adapted groups such as Actinobacteria. Nevertheless, such compositional changes do not guarantee functional stability. Climate-driven modifications can disrupt interaction networks and decouple coordinated mechanism such as carbon mineralization and nutrient release, with outcomes in systems already operating close to physiological limits (Trivedi et al., 2020; Trivedi et al., 2022). Microbiome assembly and function are further impacted by altered precipitation timing. Soil moisture pulses are closely linked to microbial functions including nutrient mineralization, nitrogen fixation, and enzyme activity. Climate-driven variations in pulse timing can disrupt the synchronization between microbial activity and plant needs. Benefits may be lost by volatilization, leaching, or immobilization when nutrient release takes place outside of plant uptake times (Van der Putten et al., 2013).

Shorter or less predictable wet windows may also bound colonization possibilities, improving the importance of microbes already exist in protected niches such as rhizosheaths, biofilms, and endospheres. While this may increase host control over microbiome composition, it also strengthen vulnerability to disturbance and loss of important taxa (Van der Putten et al., 2013). Climate impacts are significantly modulated by land-use change, which often serves as a more direct cause of microbial disruption than climate alone. Overgrazing, mining, extensive agriculture, irrigation expansion, and urbanization disturb soil structure, decrease vegetation cover, and directly damage microbial habitats. Disturbed dryland soils consistently show decreased microbial biomass, lower functional variation, and changed nutrient cycling, constraining plant establishment and recovery (Vandenkoornhuyse et al., 2015; Veach et al., 2019).

Although irrigation might alleviate local water scarcity, it may also introduce salinity, change nutrient profiles, and replace stress-adapted microbial communities with assemblages optimized for high-input systems, leaving behind land-use legacies that influence plant–microbe interactions in the future. These land-use and climate pressures interact in feedback loops characteristic of desertification. Loss of vegetation and biocrusts reduces soil stability, enhances dust emissions and erosion, and further degrades microbial communities, reinforcing positive feedbacks that weaken nutrient cycling and soil aggregation (Veach et al., 2019). Once degradation crosses thresholds, microbial communities may exist but with decreased functional capacity, limiting their capability to support plant growth even if beneficial taxa remain present. On the other hand, stabilizing microbial habitats can slow or reverse these trajectories, highlighting that microbial benefits cannot be assessed independently of system status.

## 7 Developing microbiome strategies for dry systems: from ecological knowledge to application

In light of these environmental and climate-driven constraints, microbial establishment and persistence in drylands are restricted by strong abiotic filters, spatial heterogeneity, and temporal variability, posing significant challenges for translating microbiome science into agricultural and restoration practices. However, these same constraints make drylands a crucial testing ground for ecologically based microbiome management. Syntheses progressively highlight that successful translation depends on matching microbial communities to ecological filters imposed by climate, soil, and host plants, instead of presuming universal efficacy of plant growth–promoting characteristics (Veach et al., 2019; Vílchez et al., 2016). In water-limited environment, this matching must put stability throughout drought pulses rather than maximal growth under ideal conditions. Dryland studies have repeatedly shown that ecological mismatch, rather than a lack of microbial potential, is frequently the cause of inoculant failure. Microbes chosen under mild laboratory circumstances may fail under desiccation, UV exposure, temperature extremes, or competition with resident communities (Table 1). On the other hand, even at low growth rates, microbes from arid soils, rhizosheaths, biocrusts, or desert plant endospheres sometimes have stress-adapted characteristics that encourage establishment and persistence (Vorholt, 2012). Growing evidence increasingly favors community-level strategies over single strains. While individual microbes with defined characteristics (e.g., IAA production, ACC deaminase) offer mechanistic insight, synthetic microbial communities (SynComs) combining complementary functions frequently provide greater stability and functional breadth under aridity (Wagner et al., 2016).

**Table 1:** Microbial responses and interaction dynamics across environmental stress gradients in dryland systems.

Stress factor	Typical range (drylands)	Environmental constraint	Microbial response	Functional outcome	References
Soil moisture (water potential)	−0.5 to < −5 MPa	Desiccation, limited diffusion	Dormancy, EPS production	Water retention	Alsharif et al. (2020), Bulgarelli et al. (2013), and Naylor and Coleman-Derr (2018)
Salinity (EC)	4–20 dS m^−1^	Osmotic stress, ion toxicity	Osmolytes, ion pumps	community shift toward halotolerant taxa	Aslam et al. (2022) and Singh et al. (2022)
Temperature	30–60 °C (surface soils)	Enzyme instability	Heat-tolerant taxa	Metabolic resilience	Delgado-Baquerizo et al. (2018) and Trivedi et al. (2022)
Soil pH (acidification)	pH 4–6 (localized)	Nutrient solubility, metal toxicity	Shift toward acid-tolerant taxa; reduced bacterial diversity	Altered nutrient cycling and enzyme activity	Liu et al. (2025)
Organic carbon	<1% (typical dry soils)	Energy limitation	Slow growth, metabolic flexibility	Low microbial biomass and activity	Delgado-Baquerizo et al. (2018) and Tariq et al. (2024)
Nutrient availability (N, P)	Often limiting	Restricted diffusion	Enzyme production, scavenging	Strong dependence on pulse events	Li et al. (2024) and Liu et al. (2024)
UV radiation	High	DNA damage	Pigment production, repair systems	Stress resistance	Lan et al. (2024) and Rodriguez-Caballero et al. (2018)
Soil heterogeneity	Patchy (fertility islands)	Spatial variability	Niche differentiation	Functional diversity	De Vries et al. (2020) and Philippot et al. (2013)
Hydration pulses	Episodic rainfall	Temporal instability	Rapid activation	Rapid but transient microbial activity	Alsharif et al. (2020) and Costa et al. (2018)

Effective consortia usually integrate EPS and biofilm producers (water retention), signaling modulators, nutrient mobilizers, and stress-tolerant taxa capable of persisting through desiccation. Studies on drought-responsive microbiomes have shown that functional redundancy within these communities can act as a buffer against environmental fluctuation (Ridolfi et al., 2008; Wang Y. et al., 2025). Nevertheless, complexity alone is deficient; hostile interactions or inadequate establishment can compromise performance, highlighting the need for iterative design and validation grounded in ecological theory. In drylands, microbial survival during storage and following application depends on formulation techniques such as desiccation-tolerant carriers, seed coatings and protective matrices, that position microbes near developing roots.

Seed-based distribution is especially effective because it synchronizes microbial existence with early root development and rainfall pulses (Yuan et al., 2018). Aligning application with predictable wet periods further enhances colonization success, reflecting the pulse-driven nature of dryland biology. Additionally, microbiome interventions must also be incorporated with soil and crop management stratgies that sustain habitats. Indirectly promoting microbial persistence, conservation tillage, residue retention, and mixed cropping prevent erosion, safeguard biocrust remains, and preserve fertility islands (Yuan et al., 2018). Crop genotypes different in root architecture, stress signaling, and exudate chemistry, influencing microbial recruitment; choosing or breeding plants compatible with stress-adapted microbiomes exhibits an unexplored possibility (Raaijmakers and Mazzola, 2012; Zhao et al., 2024). Biosafety and risk considerations are crucial. Introducing non-native microbes may disrupt specialized dryland communities or cause unintended outcomes, especially given sluggish recovery rates. A precautionary strategy supports local or climatically matched source communities and long-term, multi-season field testing to identify trade-offs and ensure durable benefits (Zhang et al., 2018).

### 7.1 Soil pH as a chemical regulator of environmental filtering in drylands

Environmental filtering in drylands is mainly structured by water limitation, salinity, and temperature extremes; nevertheless, these filters operate through underlying soil chemical conditions that regulate microbial activity and resource availability. Among these, soil pH plays a fundamental role in shaping microscale chemical environments by controlling ion balance, nutrient solubility, and enzyme-mediated processes. In dryland systems, where biological activity is constrained to short hydration pulses and diffusion is not high, even moderate fluctuation in pH can significantly influence how microbial communities access and transform resources (Table 1). Hence, rather than acting as an independent driver, soil pH functions as a chemical regulator that modulates the effectiveness of aridity-driven processes and contributes to the overall strength of environmental filtering. Across terrestrial ecosystems, soil pH is very crucial predictors of microbial community composition and functional potential, frequently exceeding the influence of other soil physicochemical characteristics, and in dryland systems, this control works in concert with pulse-driven hydrological constraints to shape microbiome assembly and function (Philippot et al., 2024; Coleine et al., 2024; Eldridge et al., 2018). This emphasizes the necessity of taking into account both the existence of environmental filters and the ways in which chemical conditions control microbial activity in arid environments.

### 7.2 Mechanistic coupling of pH, resource chemistry, and pulse-driven microbial activity

The mechanistic value of soil pH in drylands arises from its tight coupling with nutrient chemistry and pulse-driven microbial metabolism. Under acidic conditions, increased solubility of metals such as Fe^3+^ and Al^3+^ can enforce toxicity constraints, while P availability is often decreased due to fixation or changes in nutrient binding to soil particles (Philippot et al., 2024; Coleine et al., 2024). These effects directly influence microbial metabolic efficiency and enzyme activity, particularly during brief hydration pulses that characterize biological activity in drylands. Because microbial processes such as N transformation, C mineralization, and P mobilization are temporally constrained to brief wet periods, any pH-driven chemical limitation can disproportionately affect functional outputs. Experimental and synthesis studies exhibit that soil pH modulate microbial carbon-use efficiency, enzyme activity profiles, and nutrient cycling pathways, thereby influencing how effectively microbial communities convert transient resource availability into plant-accessible forms (Philippot et al., 2024; Coleine et al., 2024; Liu et al., 2025).

Significantly, pH effects in drylands are amplified by limited diffusion under low moisture conditions. During drying periods, decreased connectivity among soil pores limit solute motility, generating steep microscale chemical gradients. Under these conditions, microbial communities depend on localized mechanisms—including EPS-mediated retention of solutes and biofilm formation—to sustain functional activity. Acidification can disrupt these microscale processes by changing ion balance and weakening the stability of enzyme-mediated reactions, thereby decreasing the efficiency of nutrient transformation during crucial pulse windows (Liu et al., 2025). This highlights that pH effects are not only chemical but also spatial and hydrological, operating through the same microhabitat-scale processes that underpin dryland microbiome function.

### 7.3 Effects on microbial interactions and community-level stability under combined stress

Integrating soil pH into dryland frameworks further reveals that microbial interaction networks function under combined environmental constraints, where community stability is determined by both chemical and hydrological filters. Under acidified circumstances, decreased microbial diversity and shifts in dominant taxa can change network structure, potentially reducing redundancy and increasing dependence on a narrower set of stress-adapted organisms (Luan et al., 2023). Reduced microbial diversity and changes in dominant taxa might alter the structure of networks under acidified conditions, thereby decreasing redundancy and increasing reliance on a smaller group of species that have adapted to stress. Additional pH stress may increase reliance on facilitative interactions such cross-feeding, metabolic complementation, and shared EPS synthesis because dryland microbiomes already function under strong environmental filtration. Network-based studies exhibit that microbial stability under stress is closely connected to interaction structure, with cooperative networks buffering environmental fluctuations more effectively than loosely linked communities (Luan et al., 2023; Kim and Or, 2019).

In the dryland systems represented throughout this study—especially rhizosheaths, biocrusts, and fertility islands—these interaction-mediated mechanisms are necessary for nutrient retention, maintaining microscale hydration, and root–soil connectivity. The addition of pH fluctuation introduces another limitation that can reshape these interactions by modifying resource gradients and chemical microenvironments. In response, functional stability in acid-affected drylands is not determined only by individual microbial traits but by the capacity of communities to rearrange interaction networks under overlapping stressors. This supports the crucial framework of this study that interaction ecology is the primary mechanism linking microbiome structure to plant performance under harsh environments.

### 7.4 Implications for microbiome design under multi-stressor dryland conditions

From an applied perspective, integrating soil pH into dryland microbiome frameworks emphasize the necessity to design microbial consortia that remain functional across spatially and chemically heterogeneous environments. Recent techniques prioritize salinity and drought resistance, pH variation introduces an additional limitation that can influence the establishment, persistence, and performance of introduced microbiomes (Table 1). In such systems, the effectiveness of microbial interventions will depend on their compatibility with soil chemical conditions, especially in environments where pH and moisture gradients coexist. This implies that microbiome design should move beyond single-stressor optimization toward selecting communities capable of maintaining functional stability across range of environmental conditions. In heterogeneous dryland landscapes, where microsites differ in both chemical and hydrological properties, successful uses are likely to depend on matching microbial consortia to site-specific conditions rather than relying on universally applicable inoculants.

Future research should therefore prioritize testing microbiome performance under combined stress scenarios that include pH variability, with an emphasis on predicting context-dependent outcomes. Such approaches will improve the reliability of microbiome-based management strategies by aligning microbial design with the multidimensional environmental filtering processes that characterize dryland ecosystems.

## 8 Knowledge gaps, research priorities, and the path forward under global change

Despite advancements in multi-omics and sequencing, many evidence relating microbiomes to plant performance in arid conditions remains correlational. A fundamental priority is causal validation, integrating community profiling with SynCom experiments, culturing, and manipulative field investigations that directly test microbial functions (Li et al., 2025; Raaijmakers and Mazzola, 2012). Drylands provide opportunities for such work through irrigation experiments, natural rainfall gradients, and long-term observatories. There is still much to learn about microbiome assembly under pulse-driven hydrology. Additionally, the role of soil pH variability as a chemical regulator interacting with pulse-driven hydrology remains insufficiently understood, especially in shaping microbial assembly and functional thresholds under combined environmental stress. This includes the need to account for site-specific soil chemical conditions, such as pH variability, when designing microbial consortia for dryland systems. Key questions integrate when recruitment happens in relation to plant development, which compartments best predict function, and how dispersal limitation and historical contingency shape trajectories. Addressing these problems needs time-resolved sampling and experiments manipulating pulse timing and magnitude.

It is equally important to scale systems from roots to landscapes. Fertility island dynamics, biocrust recovery, and rhizosheath-level processes must be linked to field-scale productivity and climate change resilience. Integrating microbiome science with ecohydrology, landscape ecology, and remote sensing will be necessary for assuming where interventions succeed (Raaijmakers and Mazzola, 2012; Zhang et al., 2024; Zhou et al., 2016). Designing resilient microbiomes for a altering climate represents a key area. Communities should be chosen for stress tolerance, functional redundancy, and interaction compatibility instead of short-term performance. Long-term field trials spanning climatic variations are indispensable for testing resilience claims. Finally, realizing microbiome potential needs alliance with practice and policy. Stakeholder participation, economic assessment, and integration with land management frameworks are essential to move from proof-of-concept to implementation. Under global change, microbial communities are vital components of dryland resilience, influencing whether plants can survive, soils remain functional, and ecosystems avoid degradation trajectories.

## 9 Conclusion

The interaction ecology of plant-associated microbiomes is a critical component of dryland ecosystem resilience. This study presents that under the harsh environmental filtering of dry regions, microbial “benefit” is defined by the ability to maintain plant stability through unpredictable stress cycles rather than simply promoting quick biomass. This review highlights that community-level traits—such as biofilm-mediated hydraulic buffering, shared EPS production, and facilitative nutrient cycling—provide a more stronger defense against desiccation compared to single-trait bacterial strains. Microbial infrastructures at the landscape scale, such as fertility islands and biological soil crusts, are important regulators of resource redistribution, microbial source pools, and patch-scale assembly trajectories that extend beyond the root–soil interface. These structures influence where and when favorable microsites persist in drylands by linking microbial ecology with ecohydrology and plant–soil feedbacks. Meanwhile, they remain extremely susceptible to climate-driven changes in precipitation regimes and land-use disturbance, underscoring the need to consider microbiomes as essential components of dryland system stability rather than as separated biological inputs.

Research on dryland microbiomes is about to advance from descriptive associations to the development of microbiome-informed production and restoration systems. Future increases in resilience and productivity are more likely to result from layered approaches that combine soil management techniques, plant characteristics, stress-adapted microbial communities, and resource redistribution at the landscape scale. These methods emphasize synchronization with pulse-driven hydrology, compatibility with ecological assembly rules, and adaptation to site-specific chemical conditions like soil pH, all of which are crucial for maintaining functional redundancy as defenses against climatic unpredictability.

Significantly, this perspective reframes interactions between plants and microbiota as foundational components of sustainable dryland systems. Dryland microbiome science can support ecosystem restoration and sustainability agendas in addition to improving agricultural performance by aligning microbiome management with broader objectives of resource-use efficiency, long-term soil function, and climate resilience. Long-term field studies, causal validation, and interdisciplinary integration across microbial ecology, plant physiology, and landscape research, will be necessary to make this transformation. Together, the ideas presented here position drylands not only as stress-constrained systems but also as powerful natural testing grounds for microbiome-based approaches that integrate ecological theory with sustainability-driven innovation. Dryland microbiomes offer both immediate opportunities to enhance plant stability under harsh environments and a foundation for future research aimed at developing resilient and protective agroecosystems in the face of global change.
